# Exploring mistreatment of women during childbirth in a peri-urban setting in Kenya: experiences and perceptions of women and healthcare providers

**DOI:** 10.1186/s12978-018-0643-z

**Published:** 2018-12-17

**Authors:** Jackline Oluoch-Aridi, Vania Smith-Oka, Ellyn Milan, Robert Dowd

**Affiliations:** 1 Regional Research Programs Manager, The Ford Program in Human Development Studies and Solidarity, University of Notre Dame, Regional Office, East Africa, P.O. Box 49675-00100, Nairobi, Kenya; 2 Department of Anthropology, University of Notre Dame, 248 Corbett Family Hall, Notre Dame, IN 46556 USA; 3 Eck Institute for Global Health, University of Notre Dame, 120 Brownson Hall, Notre Dame, IN 46556 USA; 4 The Ford Program in Human Development Studies and Solidarity, Kellogg Institute of International Affairs, University of Notre Dame, 2167 Nanovic-Jenkins Hall, Notre Dame, IN 46556 USA

**Keywords:** Maternal health, Women, Mistreatment, Healthcare workers experiences, Peri-urban, Qualitative study, Kenya

## Abstract

**Background:**

In Kenya, indirectly caused maternal deaths form a significant portion of all maternal deaths within the health system. Many of these deaths are avoidable and occur during delivery and labor. Poor quality health service has been a recurring concern among women in Kenya, with women reporting interactions with healthcare workers that are often demeaning and abusive. This paper explores the experiences and perceptions of both female patients and healthcare workers regarding mistreatment during childbirth. This study aims to provide recommendations on how dignified care can be made the norm, specifically focusing on a peri-urban setting in Kenya.

**Methods:**

The research was accomplished using qualitative research methods with focus group discussions and in depth interviews with women and healthcare workers. The aim was to gain a deeper understanding of the manifestations of mistreatment within the context of a peri-urban setting in Kenya.

**Results:**

Female patients reported different forms of mistreatment, such as verbal abuse, physical abuse, neglect, discrimination, abandonment, poor rapport and failure of the health system to uphold professional standards. The healthcare workers described a health system that was weak and fragmented with poor policy support particularly for the new free maternity services policy leading to the mistreatment of women.

**Conclusion:**

Newly formed County Governments need to provide resources for a functioning health system to ensure an enabling environment for the provision of high quality maternal health services. This process can include feedback loops with maternity clients to ensure woman-centered services. Policy makers need to strengthen oversight for the implementation of the free maternity services Community health volunteers can be trained to provide this information. Professional associations that govern the standards of quality care for healthcare workers need to address the mistreatment through retraining and norms transformation.

**Electronic supplementary material:**

The online version of this article (10.1186/s12978-018-0643-z) contains supplementary material, which is available to authorized users.

## Plain English summary

As many developing countries, Kenya has made little progress on reducing the number of maternal-related deaths within its population. Most of these deaths occur during labor and childbirth. Evidence is clear that access to facility-based deliveries and skilled birth attendance directly impacts the number of maternal deaths. However, many women are reluctant to access health facilities for childbirth services because of the type of treatment they encounter at many health facilities. Previous research in Kenya suggests that women are subject to mistreatment at health facilities, including physical abuse such as slapping and pinching, verbal abuse such as yelling, as well as other acts of abuse and negligence. In this study, qualitative research methods were used to explore both women and healthcare providers’ experiences and perceptions of mistreatment of women during childbirth in the peri-urban setting of Dandora in Nairobi. The study found that mistreatment during facility-based delivery is widespread and is largely driven by weak maternal healthcare policies and poor rapport between different actors within the system, such as mothers, nurses, midwives, doctors, hospital administrators, and the larger healthcare system. It is necessary for the newly formed county governments in Kenya along with policy workers to seek solutions to mistreatment through provision of resources for maternity wards such as retraining as well as health systems improvements such as increasing the numbers of staff and providing adequate resources.

### Background

Maternal mortality remains a global health challenge that promises little progress of subsiding under current conditions. Many countries have failed in efforts to reduce the rates of maternal deaths, with over 300,000 maternal deaths having occurred in developing countries in 2015 [2, 23]. Evidence suggests that a reduction of these deaths can be achieved through the use of a skilled birth attendant during delivery, facility-based delivery and ensuring access to good quality maternal health care [19, 35]. Any examination of maternal mortality must take into account whether deaths were due to direct (e.g. obstetric complications, interventions, use of incorrect treatment, etc.) or indirect causes (e.g. the presence of pre-existing conditions that developed during pregnancy and increased the risk during pregnancy) [36]. Though recent research by van den Akker et al. [33] suggests that this distinction needs to be re-examined, as it places too much emphasis on direct causes while ignoring the indirect causes that might, in fact, be more significant factors in maternal mortality (see also [20]).

Kenya has experienced little progress on maternal mortality indicators as per the MDG targets of 2015. The country’s current maternal mortality rate is 362 per 100,000 deaths [18]. Most of these deaths occur during labor and childbirth. The proportion of women delivering in a health facility in Kenya had increased from 44 to 60%; despite this increase many women in Kenya continue to shun health facilities, choosing to deliver their babies elsewhere [18]. Poor quality of care as well as fear of abuse and disrespect perpetuated by healthcare workers may be some of the reasons shaping women’s decisions [1]. The provision of high quality healthcare is central to improving maternal health services and female patients’ satisfaction. Quality of care, specifically for maternity care, needs to include several components, including being safe (minimizing risk), effective (care based on existing evidence-based guidelines), timely (making sure to reduce delays care), efficient (maximizing resource use), equitable (where everyone receives good care regardless of background), and people-centered (taking into account local needs). QoC for pregnant women and newborns in facilities requires competent and motivated human resources and the availability of essential physical resources [32].

There has been an increased scholarly attention to the context within which mistreatment arises [3, 5, 13, 24]. Some of these studies have been conducted with an aim of identifying typologies for mistreatment. Bohren et al. [6] identified seven forms of mistreatment of women during facility-based delivery in sub-Saharan African settings. These forms included, physical abuse, sexual abuse, verbal abuse, stigma and discrimination, failure to meet professional standards of care, poor rapport between female patients and providers and health systems conditions and constraints. Bradley et al. [9] elaborated on the context for mistreatment as well as the drivers of mistreatment at the meso-, micro-, and macro-level. Qualitative studies exploring perceptions and experiences of both female patients and healthcare workers in diverse sub-Saharan African countries, such as Nigeria, Guinea, and Tanzania, demonstrate that mistreatment during facility-based childbirth is not only prevalent but is a growing concern [4, 8, 22].

Research in Kenya shows that mistreatment during childbirth is also prevalent. One study using exit interviews in a small rural public hospital in Western Kenya described women’s experiences with the health system as being unsatisfactory [27]. Another study comparing thirteen health facilities estimated that disrespect occurred in at least 20% of cases [1]. An additional study by Warren et al. [34], which compared care in seven counties describe the manifestations of mistreatment of women and shows that female patients expressed frustration with a lack of confidentiality during delivery, a lack of autonomy, an abandonment by providers, and unsanitary maternity settings.

A critical policy change that was relatively new during the time of this research was a free maternity health policy initiated by a presidential decree in 2013. This policy allowed all women in Kenya to access free delivery services in all public health facilities. This policy led to a considerable shift in service delivery and expectations. The subsequent high workload also brought about by the free delivery services decree led to increases in the number of women accessing delivery services particularly in the public sector (Calhoun et al., 2018). This subsequently led to high workload with women who previously used private health facilities making trade-offs and attending already busy maternity health facilities hence frustrating already overworked healthcare workers.

Research conducted in peri-urban settings have often focused on the poor quality of services, wherein they experience higher maternal mortality rates [14, 40]. Despite the fact that Kenya has many peri-urban areas, where a large proportion of its impoverished populations live, there have been no studies of the manifestations of maternal mistreatment among its population. This is an important gap, which we aim to fill. We thus describe the manifestations of the different forms of disrespect and abuse and its connection to human dignity. Dignity is defined as health care that has understanding, compassion and empathy at its core [11]. Our ultimate goal is to explore solutions on how to improve the delivery experiences of women living in peri-urban settings and incorporate dignified healthcare practices among healthcare workers.

## Methods

### Study setting

The main objective of this study was to explore female patients’ and health care providers’ experiences and perceptions of mistreatment of female patients during childbirth. The location was the peri-urban setting of Dandora in Nairobi, Kenya. Dandora has a population of approximately 300,000 people and is situated in Embakasi-North sub-county, Nairobi. The City of Nairobi’s garbage fill is located in Dandora*;* there is significant criminal activity in the area, which is raising insecurity levels. Dandora lacks basic amenities such as clean water and garbage disposal, which leads to many health and development issues for its population. The setting was selected as a study site because it is a peri-urban setting, and thus serves to help answer questions of maternal health in these areas. Studies conducted in peri-urban settings close to Dandora have estimated maternal mortality at 700 per 100,000 [40]. Additionally because of the location’s insecurity, the area is largely understudied and the maternal health situation is not well understood.

Within Dandora there are two secondary care public health centres, four private health centres (of varying size). The two other major maternities that participants mentioned are outside of Dandora. These are referral maternities, however some of the women do go there to deliver. There is a major referral hospital, which is also outside of Dandora, near Nairobi’s city centre.

Prior to choosing the site, the research team engaged in many months of continuous community engagement sessions with the local population. These sessions were facilitated by the Dandora Human Development project (established in 2011), which is a community based organization that has worked to address the development challenges by empowering the community through facilitating community engagement and enhancing people’s dignity and sense of agency. One of the community engagement sessions identified the issue of lack of quality of maternal health services for women residing in Dandora as a key developmental challenge.

### Study participants and recruitment

The study participants included nurse-midwives, doctors, and administrators from six different health facilities within the Dandora area. Using random sampling, the women were recruited during their antenatal care services at the Holy Cross parish’s Brother Andre Medical Centre. The same system of random sampling was used to recruit healthcare workers from their workstations at the different health facilities within the Embakasi-North sub-county health office, which has contacts for healthcare workers. The healthcare workers were included to triangulate the information obtained from the female patients and to ensure a balanced view about the women’s perceived experiences. Healthcare workers who had not served in a maternity ward setting were ineligible to participate in the study.

### Data collection and management

The first phase of the study was conducted between October and December 2016. The data were collected through 46 in-depth individual (IDI) semi-structured interviews and 15 focus group discussions (FGDs) with women who had given birth during the previous five years. The second phase was conducted between January and March 2017 and was done through observations and 20 in-depth interviews with healthcare workers.

Data collection was a joint effort between the principal investigators, two research assistants, a medical officer, a graduate student, and a community mobilizer attached to the Dandora Human Development Project. The FGDs were coordinated by the first author, who is a health policy specialist and also fluent in Swahili (the local language). The interviews with the healthcare workers were conducted by the third author, a graduate student who was accompanied by a Kenyan medical doctor who helped facilitate the conversations with the healthcare workers and ensured that they did not view the interviews as an audit on their work. One of the unforeseen situations was a medical workers strike in early 2017, which created a difficulty in accessing healthcare workers at their workstations. All study participants provided informed consent through a written consent form, which they read and signed. The interviews lasted between one to one and a half hours and were audio recorded for verbatim accounts. Selected healthcare workers who were interviewed outside their work areas were reimbursed for their transport and lunch costs at a rate of 1000Ksh (10 USD). Interviews were done in English were transcribed, while the interviews that took place in Swahili were transcribed, translated, and back translated by the research assistants. The transcripts were de-identified and stored in a password-protected computer.

### Study instruments

The study employed a semi-structured discussion guide for the interviews (Additional files 1 and 2).

### Ethical considerations

Written informed consent was obtained from all study participants prior to participation. The Focus group discussions (FGDs) with the women were conducted in a safe setting away from the facility (on the grounds of the parish) where they could speak in confidence about their experiences. This location offered the requisite privacy that the women needed. To enhance additional confidentiality, all FGD participants were encouraged not to discuss each other’s opinions outside the FGD setting. The names of the respondents or any personal information were not recorded in the manuscript, so as to make it unlikely for their opinions to be linked to their identities. Research approval for the study was provided by the National Council for Research and Technology Institute (NACOSTI) in Kenya. Local ethical approval was provided by Strathmore University Institutional Review Board. Additional Ethical approval was provided by the University of Notre Dame Institutional Review Board.

### Data analysis

The data analysis was conducted by the first and third author. The approach used was thematic analysis as described by Braun and Clarke [10]. Line by line coding was done on a sub-section of the transcripts independently to obtain emerging codes inductively. The software used to organize the data prior to analysis was *Nvivo 11* [28]*.* The data was classified and organized according to emergent key themes and categories, such as different forms of abuse or neglect. A codebook was designed using these inductively emergent codes and an existing literature typology on mistreatment of women (specifically [7] work), which was adapted to the Kenyan context (see Additional file 1). This codebook expressed high-level themes and code families. The similarities and differences within the themes were highlighted and analyzed. To establish inter-coder reliability, some of the coded transcripts were reviewed by an independent author.

## Results

Two data collection methods were used during this research: (1) 15 FGDs with participating women, which included between five to seven participants, and (2) 46 individual in-depth interviews. Because of the sensitive nature of the information, some women preferred to speak about mistreatment individually and in confidence, which is why there are more than three times as many interviews as FGDs. The average age reported by the women was 30 years; the majority (74%) were married, and most of the women (91%) were Christian. In terms of tribal representation, the Luo and Kikuyu tribes were most heavily represented, with 61% claiming at least one of these. Almost 50% of participants had attained at least a secondary education, and described their job as housewife. Sixty one percent of participants had previously delivered between 2 and 3 children (see Table 1). A total of 20 healthcare workers were interviewed (6 doctors, 2 clinical officers, 8 nurses/midwives and 4 hospital administrators). Details for the demographics characteristics of the healthcare workers are presented in Table 2.

**Table 1:** Sociodemographic characteristics of the women of reproductive health interviewed through In-depth individual Interviews

Characteristic	*n* (%)
Age in years	
20–24	4 (9%)
25–29	10 (22%)
30–34	13(28%)
35–39	11(24%)
40+	8(17%)
Marital status	
Single	12(26%)
Married	34(74%)
Religion	
Christian	42 (91%)
Muslim	2 (4%)
Other/Missing	2 (4%)
Ethnicity	
Luo	16 (35%)
Kikuyu	15 (33%)
Kamba	3 (7%)
Kisii	2 (4%)
Luhya	8 (17%)
Other	2 (4%)
Education	
Primary	18(39%)
Secondary	32(50%)
Tertiary	7(4%)
None	2
Employment	
Private Sector	5 (11%)
Civil servant	1(2%)
Housewife	23(50%)
Tailor	3(7%)
Trader	7(15%)
Other	7(15%)
Number of living children	
0–1	5(11%)
2–3	28(61%)
4–5	10(22%)
5 and above	3 (7%)

**Table 2:** Sociodemographic characteristics of healthcare workers interviewed through In-depth individual Interviews

Characteristics of healthcare workers				
	Doctors(*n* = 6)	Nurses(*n* = 8)	Clinical Officers (*n* = 2)	Hospital Administrators (*n* = 4)
Age in years				
20–29	4	1	1	0
30–39	2	4	1	3
40–49	0	2	0	0
50+	0	1	0	1
Marital Status				
Single	6	2	1	0
Married	0	6	1	4
Gender				
Female	3	6	0	4
Male	3	2	2	0
Years of Experience				
0–4	6	3	2	0
5–9	0	0	0	2
10–15	0	3	0	1
15+	0	2	0	1
Type of health facility				
Private	0	5	1	4
Public	6	3	1	0

### Mistreatment experiences of women

The following presents the emergent themes from the data collected on the female patients’ and healthcare worker’s perceptions of mistreatment. As was mentioned in the introduction, Bohren et al. [7] identified the following typology of mistreatment: Physical abuse, sexual abuse, verbal abuse, stigma and discrimination, poor rapport between healthcare workers and women, failure to meet professional standards and health systems constraints Our data fell within all the six major themes aforementioned with the exception of sexual abuse. Because of the prevalent stigma around the issue of sexual abuse among most populations in Kenya, participants did not mention its manifestations to us. Additionally, because of the prevailing power imbalance between healthcare workers (who are often of higher socioeconomic status than the women) it is unlikely that the women would report any instances of sexual abuse.

#### Physical abuse

The women reported physical abuse occurring during their delivery. The physical abuse was often described in terms of beatings and slaps. One woman described her experience as below;

*“…At this public hospital the doctors are usually nice, they walk around the wards and when they find you seated, they encourage you to walk. The only problem there is when you disturb them during the delivery process they beat you up, a slap will just land on you or they could beat you up with a plank of wood…” (Woman, age public hospital).*

The women we spoke with experienced fear, panic, a sense of helplessness, bitterness, and psychological pain as a result of the beatings meted out by the healthcare workers. Another woman interviewed described some of the harsh treatments she was subject to;

*“…I could see how people were giving birth and the doctors were harsh, beating people seriously and you were next and you don’t even know what you were are going to do…” (Woman, aged 23, FGD, delivered in public hospital).*

The female participants explained that they knew that the nurses were slapping them in order to assist them in their delivery of their babies, but also noted that they were never given explicit reasons for why the beating was necessary, leaving them confused and perplexed. This abuse was especially shocking for first time mothers who admitted having bitter feelings towards the nurses after these experiences, and thus considered never returning to the health facilities for any future delivery.

Our data show that there were conflicting views from healthcare workers regarding the physical abuse. When asked, the nurses particularly justified the slaps as a tool of obtaining compliance and cooperation from the women during the pushing phase of birth, citing the urgency required to save the lives of a mother and baby during the active phase of childbirth. A few of the midwives justified the physical abuse by citing their training, saying they were taught to be ‘firm’ with the women. They thus learned to obtain compliance through physical means, with the end goal of ensuring good health outcomes for the women and their babies. Nurses and midwives described their patients’ lack of cooperation and compliance, especially during labor put the women at risk of losing their lives and harming their child during a critical time as illustrated below:

*“…You know some mothers are uncooperative because of the pain you find a woman if you are so gentle with them they become unruly but if you are so gentle and so you can find some of them even injuring the baby so you have to be ‘firm’…” (IDI, Nurse-Midwife at private health facility)*

The nurse-midwives interviewed used terms such as “uncooperative”, “unruly”, “wild” and “rude” to describe some of the women. They also described a special class of women that they labeled “difficult women.” These women tended to be either underage women with no children, older women who were multiparous, or women who had not attended antenatal care services. They described these women as woefully unprepared for the eventualities surrounding childbirth. One doctor interviewed mentioned that he had encountered some very young women who were likely victims of sexual assault. He said they were extremely hostile and were hence at risk of verbal or physical abuse because of the perceived lack of compliance to medical instructions during labor and childbirth.

Almost all the different kinds of healthcare workers attempted to justify physical abuse. They stated that because female patients in labor were undergoing intense pain, their emotional state was questionable. They went on to add that these patients were unable to comply with the rigorous instructions issued, and hence the healthcare workers were forced to become physical with them. This kind of justification of physical abuse represents a clear illustration of the normalization of physical abuse during childbirth.

### Verbal abuse

Female participants described how healthcare workers attempted to silence them from asking about their own care during labor and delivery. They mentioned that the nurse-midwives predominantly asserted their power over them by using sarcastic words when they asked questions related to their care. The women also complained that the nurses castigated them for their sexuality and told them they were not present when the women were conceiving and so the women should not expect help from them. The nurses were reported to have regularly used abusive language, especially during the process of labor to suppress the noises women made during pushing. Interestingly, women were also castigated during seemingly mundane tasks, such as when they made their beds, as illustrated by the quotes below;

*“…In the wards nurses and workers were shouting at mothers, if they notice that you have soiled the bed sheets you are in trouble; that is the time you are told to wake up quickly and bathe with cold water; and at that time you are weak and can only move slowly…” (Woman, aged 26, FGD, delivered in public hospital).*

*“…I was in labor pains for three days and the nurses were saying that my cervix had not opened and eventually an artificial rupture of membranes was conducted by the nurses who were assisting me… They looked at me with contempt and used abusive language when I was pushing the baby… They could tell me to push or don’t push that really confused me. After a long struggle I told them to take me for an operation because I thought I could not manage [and] the head of the baby was already out. I was terrified to see the nurses hold my legs and forcing me to push the baby, because they had sensed danger. Eventually my baby was safe but I didn’t like how I was handled…” (Woman, aged 24, FGD, delivered in major public maternity hospital).*

Almost half of all women in the focus group discussions used terms such as “they are very harsh”, “they insult you”, “they were rude”, “they can’t handle someone in a good way”, “they look down upon you” and “they abuse you”. They claimed that the nurses abused them verbally for the most menial transgressions, for instance, the clothes they wore. They also said that they used insinuations surrounding their sexuality. These verbal abuses illustrated a power imbalance between the nurses and the women, which seemed to normalize the unwarranted verbal abuse.

*“…Sometimes maybe a mother has not been attending clinic, she has come with nothing because you have to conduct an ANC profile and know her HIV status. Some are just like they have not gotten any health education outside there. And also primiparous women do not attend clinic.so we have to shout at them…” (IDI, Clinical officer at private facility).*

### Stigma and discrimination

Stigma and discrimination appear in the following ways: (1) Discrimination based on age; (2) As ethnic-based discrimination; (3) Structural discrimination based on continuity of care from prenatal to post-natal period; 4) Discrimination based on parity; 5) Discrimination based on disease status; and 6) Discrimination based on socioeconomic status. Some women stated that if they were young, the healthcare workers would mistreat them for being involved in sexual relations and having babies prematurely. Healthcare workers would thus neglect them during labor or deny them care altogether. Other women described discrimination due to their ethnic background. They reported that they perceived that women of “higher” ethnicities were given preferential treatment over those of “lower” ethnic groups, as described by one woman,

*“…. One looks at a card and sees this is Jane and this is Mary, they would call Jane first (because her name indicates her ethnicity) and she gets attended to with priority and Mary will stay there until at night someone complains (about the queue jumping) and only then do they use the order (in which the patients arrived). This is not right as we are one tribe, just one tribe…” (Woman, aged 30, FGD, delivered at Major Referral Hospital A).*

Another unexpected dimension of discrimination was based on continuity of care from antenatal care services to delivery care. That is, women who received their antenatal services at the hospital where they were intended to deliver were given preferential treatment in comparison to those who had not.

*“…They shouldn’t dwell on where one has gone to the antenatal clinic; they get to attend to those who went to private hospitals first and leave those who have been going to the city council hospital. For example at [major maternity Hospital A], those that used to attend city council’s clinic were attend to first while those who were attending private clinics were put aside and they could actually die from there…” (Woman, aged 20, FGD, delivered at a private hospital)*

As with both verbal and physical abuse, multiparous women were also discriminated against. That is, they experienced neglect because healthcare workers assumed that their multiple experiences giving birth gave them the ability to know what to do with their current birth. As has been noted by other scholars, women with a certain disease status (specifically HIV+) experienced discrimination. They reported in confidence that healthcare workers did not want to handle them or would embarrass them by loudly calling out their disease status in the presence of other women.

### Poor rapport between women and healthcare workers

As can be imagined based on the narratives described thus far, for many women, rapport between them and their providers was a major challenge. The women described a lack of supportive care as well as inattentiveness and insensitivity regarding their experiences of pain. They stated that they felt that healthcare workers deliberately ignored their plight. When women attempted to call the doctors they were rebuked and left to deliver on their own.

*“…I tried calling the doctor who said I had just arrived [and] it wasn’t still time. … I actually forgot, [and] so I took myself to the ward and climbed into bed. In the process my water broke; they then came started making noise at me asking why I had made the place dirty. When [the nurse] left, the child came out and she just heard the child crying; the child almost slid and fell because of that water. I delivered on my own; they came and assisted me with the cutting [of the cord] but I just delivered on my own…” (Woman, aged 28, FGD, delivered in a public maternity).*

The healthcare workers reported that the women always expected to receive high quality health care services, privacy, and personalized attention, such as counseling, despite the acute shortages of healthcare workers. Participating nurses reported that female patients seemed to expect better quality service from private facilities than public health facilities. Healthcare workers also described how an increasing number of women delivering their babies expected to receive all their services for free, including miscellaneous items such as free clothes. The reason behind this was the Kenyan Government’s newly introduced policy of free maternity services at all public health facilities. Detailed instructions on implementation of this policy to healthcare workers were lacking and this lack resulted in great confusion and frustration for most healthcare workers. This underlying frustration led them to justify their many forms of mistreatment to the women.

*“…Because it’s free maternity they think they’ll walk in and walk out with everything for the baby. So most of the times when they come, they come without children’s clothes. So when they come, they find out it’s only the service that is free, everything else they’re supposed to bring for themselves. So this is usually the biggest problem most times …” (IDI, Doctor in Major Maternity Hospital A).*

The healthcare workers we interviewed felt that the women did not understand how the health system worked and were therefore imposing their expectations for a high quality care. Despite the fact that this was a heavily constrained health system (which, as was stated earlier, was undergoing a strike) patients continued to demand good quality services and personalized care. The health workers in response to their female patients’ expectations deliberately failed to build a rapport with them and would be rude towards them. The healthcare workers would mistreat them as a way of expressing discontent with the women’s attitude towards the free delivery services.

### Failure to meet professional standards

Women described feeling neglected and that their care was abandoned at critical moments during the delivery due to healthcare workers claiming that they had other competing demands. One woman in a focus group stated that:

*“…I called the doctor when I began feeling the baby coming down, but the doctor refused to come and so I delivered on my own. And that is when the doctor was called by the other people. When the doctor came he cut the umbilical cord and before the doctor could attend to me there was another woman nearby also giving birth. The doctor left my child uncovered and unattended and went to the other woman and came back after she had delivered and took my child to the nursery…” (Woman, aged 23, FGD, delivered in a private health facility).*

What is interesting about this above description is that the doctor does, indeed, have other competing claims. While we hear the concern from the patient herself, who felt abandoned, we can also identify how the doctor was trying to attend to two patients at once, given the shortage of workers and time constraints. The women emphasized a dearth of supervision of deliveries because of the shortage of staff and the work demands of the busy maternity sections. They described women being left with trainee clinicians who were often inexperienced and would make mistakes. The women construed these mistakes as deliberate mistreatment, even though it could be argued that the doctors were trying to manage an almost impossible situation as best they could.

The women described that a few healthcare workers were clearly inebriated and were responsible for enacting physical violence on their patients, as described below.

*“…At this point there were only two trainees in the ward, one doctor was drunk and was sleeping, when the trainee gets stranded with anything. He comes and when you try to argue with him he beats you up. When I was about to start pushing the baby, the trainee came and was unable to help me. When the doctor came he painfully pressed my stomach and I told him he was hurting me and he said that this was not his work ….His work was to deliver the baby and asked if I hadn’t called for him; but I gave birth well…” (Woman, aged 27, FGD, delivered in Major Referral hospital).*

In the above narrative there are two healthcare workers, one drunk and unable to manage the work, and another who enacted what was likely the Kristeller maneuver (or fundal pressure), consisting of pushing down on the fundus to move the baby out. This procedure is considered to be a standard, but unnecessary and very aggressive, practice within obstetrics, with many medico-legal implications [21].

Women described other unnecessary procedures, such as episiotomies, or other procedures done repeatedly by students and different healthcare workers without their consent as illustrated below.

*“…I have three kids my firstborn was born at [Maternity hospital A] but I used to go to [small clinic B] where I had high blood pressure so it forced me to attend clinic weekly. On my last weeks of pregnancy I was referred to [Maternity hospital A] where I was examined by students and the real doctors were nowhere to be found. It took me around two hours before I gave birth to a baby was weighing 2.5kg. It made me wonder why they did [an] episiotomy and they also took a lot of time to stitch me. The reasons I was given was that they were waiting for the higher ranked doctors who supervise them to guide them on what or how they should do it. After waiting for a long time they decided to stitch [me]. Unfortunately after 15 mintues, when the doctors came, it was redone. I felt a lot of pain…” (Woman, aged 30, FGD, delivered in Maternity hospital).*

### Health systems conditions and constraints

The women mentioned that some of the mistreatment was as a direct result of poor quality of service exhibited by the overcrowding at public hospitals and acute shortages of healthcare workers. They suggested that the number of healthcare workers needed to be increased to improve the quality of care offered during delivery services. One participant stated,

*“…They should add more nurses. The existing nurses are few in and the patients are many. That’s why they were going from this side to that side and they are tired because they are also looking for a salary increment. If they have good salary they will handle people with care and if they attend to two people they don’t get tired. They should employ more nurses to avoid one nurse attending to 10 patients and to improve their services…” (Woman, aged 22, IDI, delivered at public health facility).*

Some women suggested that the lack of bed spaces available at the major hospitals for delivery services was due to the increased number of female patients accessing medical services. They added that the government could add the required space needed for the delivery beds. This deficiency in infrastructure generally led to women receiving poor quality services. For instance, women sometimes shared beds with other patients, gave birth on the floor, or were ignored and neglected by healthcare workers. Women defined these lacks as forms of mistreatment. Two women describe their experience below;

*“…The women were seated outside on the bench when one delivers. That is when the other one then gets a bed. The room was small. They could add another room because I don’t know if people are still giving birth as much. The doctors there wanted to assist but the beds were occupied. They would ask if people didn’t want to go elsewhere even though the delivery process there was free. Therefore, women had to wait by the bench; when a woman would report that they were in pain, they would be told to go home and come back the following day; they were unable [to do that] and some would sit by the bench for days. The delivery beds were few…” (Woman, aged 28, IDI, delivered in Maternity Hospital B).*

*“…I don’t know about [Major Maternity Hospital A]. I don’t know what they could add because there were a lot of people during that time. Probably they could add a room as I think they added doctors at [Major Maternity Hospital B]. The room was small; the delivery room was small at that time; it had two beds on one side and two on the opposite side. There were 4 beds in total…” (Woman, aged 34, IDI, delivered in Maternity Hospital A).*

Healthcare workers concurred with the women’s descriptions of the health facilities. They similarly described the state of the public health facilities as deplorable, citing that the capacity of the health system was lacking. They claimed that the main government maternity hospitals serving women in peri-urban settings such as Dandora lacked basic physical facilities, such as sufficient number of delivery wards, beds, and waiting rooms. They described the facilities as few and lacking in quality to meet the needs of the women accessing services. They added that there were acute shortages of basic supplies, such as gloves and surgical equipment, as well as general utilities such as electrical power and water. These latter two were frequently unavailable, which not only inconvenienced the healthcare workers, but also compromised the quality of services they could offer. This situation thus resulted in poor patient outcomes. The quote by the medical officer illustrates the issue;

*“…Sometimes you use ergometrine (medication used to cause contractions of the uterus to treat heavy vaginal bleeding after childbirth) and we don’t have sterile gloves. Sometimes you want to remove a retained placenta, you don’t have the gynecology gloves and you have to improvise. You cut up the other gloves and patch it up. Sometimes in the [operating] theatre you don’t have suturing material… are you serious? It’s something that is quite sad. Sometimes in the middle things can be running smoothly then all of a sudden there is no suture material, there is no water, just like that. There is no water, there is no electricity…” (IDI, Doctor in Major Public Maternity Hospital B).*

Healthcare workers described the maternity and labor ward settings especially in the larger referral hospitals as chaotic scenes that were routinely understaffed. Midwives described situations where they were simultaneously helping patients in labor and triaging the ones ready for delivery. They went on to say that the female patients who were perceived to be difficult were often left on their own, an action which the patients later construed as abandonment. The midwives claimed that their working environment where there was a lack of assistance made them easily prone to stress. This situation led them to lose their tempers with ‘difficult’ women more frequently. Additionally, the healthcare workers expressed frustration at their workload, with one midwife vividly describing having to regularly attend to multiple women during critical phases of labor simultaneously, leading her to prioritize only the cases that seemed most urgent in her perspective.

*“…So like I once took a patient to [Public Maternity B]… You see someone [attending to] almost 10 mothers… One mother is calling the other is also calling… In fact they even look as if they are confused. So (they try to hurry), they try to sew one while another patient in the second stage of labor is calling them and its only one nurse. So you find that this time factor makes the stress of work too much. The patient nurse ratio is, I don’t know, it is very, very low. A whole room is just attended by one person. So you just empathize [with their predicament]…” (IDI, Nurse-midwife currently at private facility).*

Another main frustration with the health system was the ineffective referral systems in most health facilities that subsequently lead to the delays in care. Women constantly interpreted this phenomenon as mistreatment in the form lack of support and attention to their needs. The healthcare workers, especially those stationed at maternity hospitals, mentioned that they frequently received women who had been referred from far-off smaller facilities who were brought in after botched deliveries. The healthcare workers said that the patients often arrived with heavy bleeding, and they were expected to attend to them despite the obvious adverse clinical consequences of late referrals.

*“…They always refer when it’s too late… There are situations where they stay with a mother who has been bleeding for quite a while, so when they see they are not going to help the mother they bring [her] here to our facility… The number one cause for most of the mothers [losing] their lives is postpartum hemorrhage… despite making the necessary interventions on time…” (IDI, Medical officer in public maternity hospital B).*

### Culture of blame

There was a prevailing culture of blame in these medical settings. Healthcare workers, particularly those at lower end of the hierarchy, strongly felt that they took the ‘direct hit’ for any adverse outcomes on the women or children. They also expressed fears at being blamed for outcomes that they largely thought were a result of non-compliance by the women. They said they were blamed by those higher up in the medical hierarchy (such as doctors). Additionally, they stated that blame was meted out at different levels and by different people. Sometimes it was doctors blaming midwives and sometimes it was the patient’s family blaming both doctors and midwives. Workers mentioned that this fear of blame is what drove them towards harsh treatment.

*“…You know generally as human beings, it’s something natural that when someone is grieving, the first person you see is the one you want to blame. […]. And as human beings we always blame the person, then ask questions later. These relatives don’t want to know and most of them don’t understand how the system works…” (IDI, Doctor at Major public Maternity B).*

*“…Makes them less cooperative (laughs) and then complicates the delivery most of the time. Psychologically they get traumatized, you can see them withdrawn and if they have a problem they won’t be free enough to ask you for help because they are not sure how you’d react to them…” (Medical officer in Public Maternity Hospital A).*

When asked why the women thought they were mistreated by healthcare workers, many didn’t seem to know the answer to the question, adding that they pleaded with healthcare workers to be patient with them during labor. They expressed fear around resuming care seeking activities at facilities where they had been mistreated and considering other options for childbirth, including home births.

Occasionally, patients and their families reported the mistreatment to medical regulatory agencies or sued healthcare workers in cases that resulted in maternal mortality. Despite this, many women expressed frustrations that there were no formal avenues set up in public and private sectors to discuss mistreatment or redress poor quality services. They added there were no feedback mechanisms at the hospitals to lodge complaints about poor quality services. Many said they were afraid of the consequences and future retaliation that they would face from the healthcare workers during subsequent visits to the health facilities if they ever took action. These fears were compounded by the distinct power and class dynamics inherent in the patient-caregiver relationship.

### Additional findings

The primary focus of this article has been to understand the ways that mistreatment in Kenyan hospitals occurs. It is important to add that there were also a few female participants who experienced positive birth experiences. They mentioned that the nurses who assisted them during the delivery process were extremely helpful. They described being accompanied throughout the entire delivery process. Several women referred to one mission hospital that consistently had high quality care. At this particular hospital, the women described the doctors and nurses as “being with you all the way” and provided detailed examples of what their positive experiences were. One woman described the experience as “relaxing” and confirmed that she gave birth to all her children at this particular hospital. She said,

*“…I had to now go to [Mission Hospital A] every other week. They kept examining me to know what the problem was. Everything was relaxing; the doctors are with you all the way; if you begin having pains they ask you what you would want; if you turn this way they apologize; they rub your back. When the baby begins to come out, they come and assist you and if you have to be stitched they do it there. They wash the baby and you also get to shower; they treat people well. I gave birth to all my children there…” (Woman, aged 25, FGD, delivered at Mission Hospital A).*

### Discussion

This qualitative study explored female patients’ and healthcare workers’ perceptions and experiences of mistreatment during labor/delivery. As other studies on mistreatment during childbirth have noted [7, 17, 23], our data shows that not only was abuse prevalent, it tended to match maternal abuse found elsewhere. Participants mentioned that the most frequent was a failure to meet professional standards, followed by verbal abuse, physical abuse, health systems constraints, and a poor rapport between healthcare workers and women; the least frequent were stigma and discrimination.

In our study lack of professional standards during delivery was identified as a major problem. We discovered that younger women, multiparous women and women who failed to attend ANC were most frequently mistreated. This pattern has been identified in similar studies assessing prevalence of disrespect and abuse that found that women under the age of 19 were more likely to be mistreated by healthcare workers (Second Author 2013; [1]). The healthcare workers participated and reinforced the culture of blame, by not only blaming the women for getting pregnant, but by also increasing the level and form of abuse (through reducing their privacy or shouting at them for their lack of knowledge of their bodies).

Verbal abuse was very prevalent in this setting; indeed, contempt and use of derogatory terms was commonplace. Verbal abuse and comments about a woman’s sexuality was mentioned by almost every female participant interviewed. The most popular rebuke mentioned was the scolding about how the women had enjoyed sexual intercourse but were now expressing pain during birth; this abuse has been identified in several different contexts, especially amongst women of lower socioeconomic status ([7, 16, 26, 39]; Second Author 2013) It is likely that, because the women in this study were from a lower socioeconomic status, the healthcare workers abused their position of power. This form of abuse was intertwined with discriminatory practices based on age, parity, socioeconomic status, antenatal care attendance, and ethnicity [31]. Our female participants felt that because of their income status, healthcare workers who were from higher socioeconomic status would mistreat them and offer them little or no guidance or explanations when providing care. This abuse arose either because of healthcare provider frustrations with their patients and the system, or was a deliberate way to exert power on populations that would not fight back. There were a few incidences of women resisting but most of them said they were afraid of being denied future services and would instead just take the verbal abuse.

As with other studies across the world, physical abuse was prevalent. What was surprising, however, was how much the women did not question the physical abuse. Several women seemed to be accepting of some forms of physical abuse, such as the slaps and pinches, stating that they were for the sake of their health and their babies during pushing. Such forms of abuse have been observed in studies within the African context [12, 22, 25]. Social norms and attitudes around such violence allow the normalization of these forms of physical abuse in this context. As Bohren [5] state in their work in Nigeria, the abuse is seen as a way of ‘rescuing’ the lives of mother/infant. The nurses in our study, at the end of a delivery, would often convince the patients that without the beatings their children would have been harmed and had adverse outcomes. Conversely to the concerns expressed by the women, the healthcare workers prided themselves on having been taught to be “firm” with their patients. We argue that it is thus important to understand and investigate the social norms of acceptability of mistreatment not only to understand how and why they occur, but also to examine them as facets arising within a gender-based violence frame [38].

Health systems constraints were mentioned by almost all the healthcare workers citing the mistreatment as unintentional and largely due to staff shortages and lack of resources. Such health constraints (including a lack of resources and facility culture) in Kenya have been identified as drivers for mistreatment [34]. Some women seemed to understand these health system constraints, and thus perceived the mistreatment as unintentional, while others claimed that the mistreatment was deliberate and intentional. However studies by Freedman and Kruk argue that despite disrespect and abuse being perceived differently by women and healthcare workers in the context of health system constraints, there are certain practices that are “unambiguously disrespectful” ([15]:e42). These practices should be identified in each context, while the social norms surrounding their acceptability needs to be challenged. We would argue that behavioral anomalies amongst healthcare workers that drive cultures of mistreatment within health institutions need to be not only addressed, but also challenged.

The poor rapport between women and healthcare workers primarily manifested as abandonment and neglect during labor and delivery. The women described several instances where the neglect led to fatal consequences. This practice has been witnessed in settings where nurse-midwives want to create social distance between themselves and the people they are serving. This situation was described by Jewkes et al. [16] in their study of nurses in a South African context. In this study there were several instances where healthcare workers, particularly nurse midwives, wanted to exert control over their patients and the childbirth process, which led to patients feeling mistreated. Avenues for redress for this situation need to be particularly created with women have access to accountability mechanisms especially to professional associations whose role is to regulate their membership.

The drivers of mistreatment in our study seemed to stem from a general perception of the women that healthcare workers and the institutions they work in fail to provide professional care. According to a framework put forward by Freedman and Kruk [15] mistreatment can manifest itself and be driven at the individual, structural, and policy level. In our study all three levels interacted to promote mistreatment. At the individual level between the healthcare workers and the women evidence pointed to a need for healthcare workers to promote abusive behavior as a way of asserting control of the women ((see, for instance, [9, 16]).. At the structural level, institutions such as health facilities, either promoted cultures that condoned mistreatment or were largely unaware of it and its manifestations. Lack of supervision of medical staff and shortages of medical staff increased this unintentional mistreatment. Other institutions such as medical schools were also implicated in this situation, as they were the places where healthcare workers learned how to interact with patients. Professional bodies governing the conduct of healthcare workers were also largely uninvolved in the regulation of their members in the mistreatment. At the policy level the new free maternity policy was a factor increasing the mistreatment due to the elevated expectations for quality maternity care simply because of its free status. Interventions that increase the understanding of the entitlements, such as deploying community health volunteers at the health facilities to inform women of the coverage package under the free maternity services, can help assist women to reduce the friction between themselves and healthcare workers regarding expectations and greatly reduce mistreatment driven by mismatched expectations.

### Limitations of the study

One main limitation of the study is the self-reporting of abuse. This method makes it difficult to detect underreporting or exaggeration, especially around a sensitive and highly subjective topic like mistreatment [29]. Additionally, because of the sensitivity of the topic, access to health facilities for observations is limited (see [30]). An important limitation was the location for the interviews (within the compound of the church within range of the health facility), which might have prevented participating women from speaking openly. Finally, the focus on only the negative experiences might be an additional limitation, as only in conversations did positive experiences of delivery emerge ((see also [34]).

### Recommendations on interventions for reducing mistreatment

We want to end this article with a discussion of some of the recommendations for reducing mistreatment. Female participants suggested that healthcare workers needed to show more compassion and patience to women in labor. They emphasized the need for a system to provide feedback on poor quality of care at public facilities. Professional societies that govern healthcare workers such as the Nursing Councils and the Kenya Medical Practitioners and Dentists Boards need to be consulted about health facilities whereby reports have been lodged regarding mistreatment of women, these institutions should strengthen accountability mechanisms and support processes for legal redress. Additionally these professional associations should seek to re-train their healthcare workers especially cadres such as nurse midwives who were predominantly involved the management of labor and childbirth. This training should include elements that address soft skills such as interpersonal relationships such as treating patients with dignity and avoidance of discrimination based on background. Previous studies in Kenya such as [34] described strategies for healthcare workers such as value clarification and attitude transformation workshops as well as stress management [34].

There is a need for greater support from the County Government that manages the public maternity hospitals in peri-urban settings where a lot of the mistreatment was described as happening. The County Government needs to provide adequate resources in terms of adequate numbers of supervised staff and supplies as well as strengthen education on the package of interventions available for free maternity service. The need The County Government needs to have a feedback mechanism for redress for complaints of mistreatment. The need for greater support is in line with guidelines that have been provided by WHO [37]. This statement also calls for Governments to be involved particularly in research as well as implementation of interventions to improve disrespect and abuse in maternity settings. The healthcare workers suggest that the county government needed to investment in health education for women particularly during antenatal care on what to expect particularly for the young first time mothers and identification of victims of sexual abuse and provision of counselling services for them.

Implementation of the relatively new government policy on maternal health services needs to be improved with supplementary health education to the women on what the included minimum package of interventions that is covered including excluded services. The implementation of the policy needs to be clarified at all levels of the health system and women need to be educated by trained community health volunteers so as to curb the unrealistic expectations described particularly by low educational status women in peri-urban settings such as the ones in this study. This will in turn help reduce common forms of mistreatment verbal abuse that results from the misunderstanding about entitlements of the policy.

## Conclusion

This study has shown that mistreatment of women during facility based delivery is rife in peri-urban settings as appreciated by the vivid descriptive accounts by both women and healthcare workers. This mistreatment is largely driven by an unbalanced relationship between healthcare workers and the women they serve due to socioeconomic status and institutional norms that normalize mistreatment. Despite the fact that there is free delivery service as a policy oversight for its implementation needs to be strengthened to clarify expectations through providing adequate information to women accessing delivery services on the coverage of services as well as exclusions to reduce misunderstandings. Medical Professional associations need to be involved in addressing the mistreatment issue amongst their members through research and creating accountability procedures. County Governments need to address mistreatment through providing resources for a functioning health system that can provide an enabling environment for the provision of high quality maternal health services and ensuring women give birth in a dignified manner.

## Additional files

Additional file 1: FGD guide for women’s interviews. (DOCX 14 kb)

Additional file 2: In depth Interview guide for healthcare workers. (DOCX 14 kb)
