# Supplementary material for: Exploring mistreatment of women during childbirth in a peri-urban setting in Kenya: experiences and perceptions of women and healthcare providers
Source: Reprod Health. 2018 Dec 17;15:209. doi: 10.1186/s12978-018-0643-z (PMC6296108; doi:10.1186/s12978-018-0643-z)
Supplement: Supplementary file 1 — FGD guide for women’s interviews. (DOCX 14 kb) [file 12978_2018_643_MOESM1_ESM.docx]

QUESTIONAIRRE FOR FOCUS GROUP DISCUSSIONS

The Focus Group Discussions are aimed towards women who have given birth within the last 5 years. Starting broad (birth story, concerns, fears, joys). Then a bit more specific (issues of obstetric violence, perhaps, complaints about caregiving, role of different clinicians or others). Then follow up with individual interviews with each woman, funneling in with these themes of caregiving, role of clinicians, fear/violence, etc.

Questions

1. Tell me about your birth story? When did you find out you were expecting a baby?
2. What were your concerns, fears and joys? Where you excited to learn that you were pregnant? Were you worried about anything?
3. Where did you attend your antenatal care services from?
4. How many times before delivery did you attend the clinic?
5. How did you like the facility that you have been going to?
6. What was the quality of care like?
7. Did you have to pay for anything e.g. the card, laboratory tests or anything else?
8. Where did you give birth? Who decided for you to give birth there?
9. Who transported you to the birth place?
10. How did you pay for the transport and admissions to the hospital?
11. How long did it take you to reach the place where you gave birth?
12. Did you have any challenges meeting the expenses related to the child birth process?
13. Who assisted you during the birthing process?
14. Did you have any complications during your delivery?
15. Did you have any fears and concerns about violence during your delivery process?
16. How were you treated by the clinicians at the place where you gave birth?
17. Did you ever receive respectful care by the health care workers, at the facility were you receive care if so explain?
18. Did you ever received bad treatment by health care workers at the facility where you receive care if so explain?
19. In what ways would you like delivery services to be improved?
20. Do you have any recommendations for the healthcare workers and the Government on how to improve maternal health services to be more respectful?
