# Supplementary material for: Exploring mistreatment of women during childbirth in a peri-urban setting in Kenya: experiences and perceptions of women and healthcare providers
Source: Reprod Health. 2018 Dec 17;15:209. doi: 10.1186/s12978-018-0643-z (PMC6296108; doi:10.1186/s12978-018-0643-z)
Supplement: Supplementary file 2 — In depth Interview guide for healthcare workers. (DOCX 14 kb) [file 12978_2018_643_MOESM2_ESM.docx]

DISCUSSION GUIDE FOR INDEPTH INDIVIDUAL INTERVIEWS WITH HEALTH WORKERS & HOSPITAL ADMINISTRATORS

The In depth Individual interview are aimed towards health workers who have worked in a maternity ward previously (in the past 5 years). They could either be specialist obstetrician /gynecologists, medical officers, clinical officers, midwives and nurses. This discussion guide explores the experience of motherhood for women residing in Dandora and addresses specific issues such as mistreatment during delivery, drivers and deterrents of mistreatment during delivery, social norms and acceptability of mistreatment during delivery services, quality of care within the maternity systems, the role of clinicians during delivery the impact of mistreatment on care-seeking behavior and health system factors affecting women’s birth experiences in Dandora.

The health workers and administrator will also be asked about what they think are recommendations to Government and private facilities that can contribute towards improving the care given to women during delivery

Discussion Themes

1. For how long have you worked at a maternity ward? How many child births have you experienced roughly? What are the experiences the women you serve reporting as had at the facility where you work
2. Have you witnessed any of your colleagues treating women well during child birth? Mistreating women during the intrapartum phase? Kindly provide Examples that you or your colleagues have witnessed.

1. What are some of the expectations for care that he women who come to your maternity clinic report? How do they express they would they like to be cared for?

1. What are some of the challenges you have experienced working at the maternity ward?
2. What are some of the decision making processes that women coming to deliver at your facility have to contend with? Are they supportive? Unsupportive?

1. What do you think are the factors that lead to women been mistreated during maternal health services such as ANC and childbirth?
2. What do you think is the impact of women been mistreated during maternal health services such as ANC and childbirth?

1. Do you have any suggestions/recommendation to health care workers and the Government on how facility based delivery experiences can be improved?
